# Human resources for supply chain management online discussion. What do the countries have to say?

**DOI:** 10.1186/2052-3211-7-S1-P5

**Published:** 2014-12-17

**Authors:** Andrew N Brown, Erin Hasselberg, Pamela Steele

**Affiliations:** 1 People that Deliver, Copenhagen, Denmark; 2 John Snow Inc., Boston, MA., USA; 3 Pamela Steele Associates (PSA) Ltd, Oxford, UK

## Background

PtD brings together a range of global stakeholders with expertise in SCM with a mission “to build global and national capacity to implement evidence-based approaches to plan, finance, develop, support, and retain the national workforces needed for the effective, efficient, and sustainable management of health supply chains.” To ensure PtD continues to understand country experiences, an online discussion via the Independent Association of Public Health Logisticians (IAPHL) was designed to interact with a large variety of countries globally.

## Method

Under the theme of ‘Systematic Approaches to Human Resources for Health Supply Chains’ three content experts were asked to prepare a two page evidenced brief addressing one of three sub themes. Over a four week period (April – May 2014), each brief was presented to IAPHL members and seeding questions used to promote asynchronous discussion. Moderators engaged in the discussion and used a process of thematic analysis to assess the discussion.

## Results

103 contributions were made (Av. of 9 [1-17] contributors per question), 24 countries were represented (av. of 7 (1-12) per question). Several sub themes emerged from the three topics:

HR as a barrier. A lack of supply chain strategy and unclear patterns in decision making responsibilities dominated, with an underestimation of the SC managerial competencies required.

A systematic approach. A need for SCM champions and medium-to-long term HR and SCM strategies was clear, with professionalization of the SCM workforce identified as the most significant challenge.

Education and continual professional development. Pre service education was seen as an early foundation that must be built on by competency based in-service training. A lack of resources was seen as the main barrier.

## Discussion

It is clear that HR issues are a barrier to the effective running of health supply chains in many countries. Improving the professionalization of health supply chain cadres is seen as a priority by a number of countries with appropriate combinations of pre-service foundation training and competency based in-service training called for. Country based support is required to allow governments to systematically assess HR aspects of their supply chains while competent health supply chain leaders are needed to enable improvement plans to be successfully implemented.

## Lessons learned

Issues concerning HR for SCM exist across a range of countries. The IAPHL discussion platform proved to be an effective forum to engage a variety of country based stakeholders concerning issues around HR for SCM.

